# Surprises from a boron-rich semiconductor under pressure

**DOI:** 10.1093/nsr/nwag137

**Published:** 2026-03-09

**Authors:** Artem R Oganov

**Affiliations:** Skolkovo Institute of Science and Technology, Russia

Boron-rich semiconductors are an emerging class of materials known for their exceptional mechanical hardness and thermal stability, making them promising for applications in extreme environments [1–3]. Their optoelectronic properties under ambient conditions have been studied, but their performance under high pressure typically degrades due to bandgap narrowing and eventual closure—the well-known Wilson transition. In a new paper, however, the Xiang-Feng Zhou’s group and collaborators [4] have discovered that a specific boron-rich semiconductor exhibits remarkable and counterintuitive pressure-driven enhancement of its optoelectronic performance.

In conventional semiconductors, applied pressure increases orbital hybridization, which narrows the electronic bandgap, often leading to increased dark current and reduced device efficiency [5]. This fundamental limitation hinders the development of robust optoelectronic devices for high-pressure environments, such as deep-Earth sensing or aerospace. A highly desirable but rare phenomenon is the so-called anti-Wilson effect, in which pressure causes the bandgap to widen, thereby suppressing the dark current and potentially improving the photoresponse.

**Figure 1.**
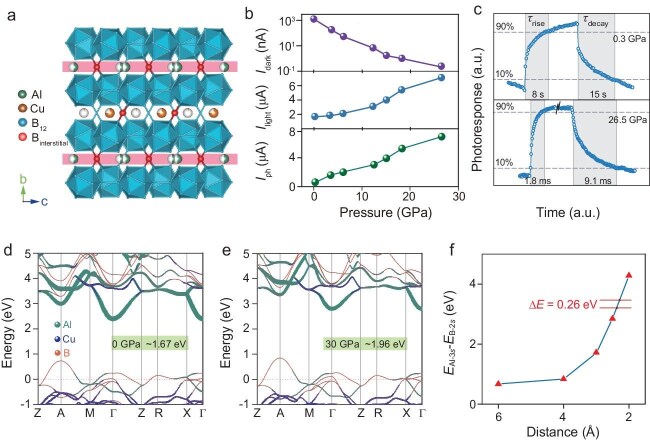
(a) Crystal structure of AlCu_1−δ_B_25_ viewed along the [100] direction. (b) Pressure-dependent *I*_dark_, *I*_light_ and *I*_ph_ curves. (c) Transient photoresponse curves at 0.3 GPa and 26.5 GPa. (d and e) Orbital-resolved band structures at 0 and 30 GPa, respectively. (f) Energy separation values as a function of interatomic distance between Al and B, based on the two-atomic system.

Zhou and co-workers hypothesized that the unique electronic structure of certain boron-rich frameworks could host such an anomalous effect. To test this, they synthesized high-quality single crystals of the semiconductor AlCu_1__–__δ_B_25_ (Fig. 1a). While its high hardness (∼30.4 GPa) and thermal stability (>1400°C) provided the necessary mechanical robustness, its electronic response to pressure was unknown. Zhou and colleagues measured the optoelectronic properties of devices based on this material under pressures of ≤26.5 GPa by using a diamond anvil cell. The current-versus-pressure curves presented in Fig. 1b show that the photocurrent increased by >20-fold, while the dark current was suppressed by nearly four orders of magnitude. This synergy resulted in an unprecedented improvement in the on/off ratio of >10^5^-fold. Simultaneously, the response time accelerated by three orders of magnitude (Fig. 1c). The combination of structural stability

with this giant anti-Wilson effect leads to performance metrics, such as a detectivity (D*) of ∼8.59 × 10^13^ Jones at 26.5 GPa, which are orders of magnitude higher than the initial values. These properties are visualized in the stark contrast between the slow, weak response at low pressure and the fast, strong response at high pressure.

The remarkable fundamental results obtained by Zhou and co-workers on the origins of the anti-Wilson effect in AlCu_1__–__δ_B_25_ are explained through first-principles calculations. They identified that the pressure-induced widening of the bandgap arises from an upward shift in the conduction band minimum, dominated by Al-3*s* states, due to interaction with B-2*s* electrons (Fig. 1d–f). This discovery not only provides a novel mechanism for property optimization, but also establishes a path for designing durable electronics for extreme conditions. The work successfully translates this insight into a proof-of-concept for a high-pressure-hardened photodetector.
